# Decision-Making in Suicidal Behavior: The Protective Role of Loss Aversion

**DOI:** 10.3389/fpsyt.2018.00116

**Published:** 2018-04-05

**Authors:** Gergö Hadlaczky, Sebastian Hökby, Anahit Mkrtchian, Danuta Wasserman, Judit Balazs, Núria Machín, Marco Sarchiapone, Merike Sisask, Vladimir Carli

**Affiliations:** ^1^National Centre for Suicide Research and Prevention of Mental Ill-Health, Department of Learning, Informatics, Management and Ethics, Karolinska Institutet, Stockholm, Sweden; ^2^Vadaskert Child Psychiatry Hospital and Outpatient Clinic, Budapest, Hungary; ^3^Institute of Psychology, Eötövös Loránd University, Budapest, Hungary; ^4^Skylark Health Research Ltd., London, United Kingdom; ^5^Department of Medicine and Health Science, University of Molise, Campobasso, Italy; ^6^National Institute for Health, Migration and Poverty, Rome, Italy; ^7^Kazakh National Medical University, Almaty, Kazakhstan; ^8^Estonian-Swedish Mental Health and Suicidology Institute, Tallinn, Estonia; ^9^School of Governance, Law and Society, Tallinn University, Tallinn, Estonia

**Keywords:** loss aversion, decision-making, suicide, attempted, mental health, suicidal ideation

## Abstract

**Background:**

Loss aversion is a central and well operationalized trait behavior that describes the tendency for humans to strongly prefer avoiding losses to making equivalent gains. Human decision-making is thus biased toward safer choices.

**Aim:**

The aim of this study was to explore the relationship between loss aversion and suicidal behavior in a large cohort of adolescents recruited in 30 schools of seven European countries for a longitudinal study (Current Controlled Trials ISRCTN65120704). We hypothesized that individuals with higher loss aversion would be less likely to attempt suicide.

**Methods:**

A mixed monetary gamble task was used to generate loss aversion scores for each participant. Logistic regression was used to estimate the cross-sectional association between loss aversion and life-time suicide attempts in the baseline sample (*N* = 2,158; 156 attempters), and incident attempts were predicted in a 4-month prospective model (*N* = 1,763; 75 attempters). Multiple regression was used to estimate the association between loss aversion and suicidal ideation.

**Results:**

Loss aversion was a significant predictor of attempted suicide in both the cross-sectional (OR = 0.79; *P* = 0.005) and prospective analysis (OR = 0.81; *P* = 0.040), adjusting for depression, anxiety, stress, and sex. The correlation between pre and post measures of loss aversion was *r* = 0.52 (*P* < 0.001). Interestingly, although depression, anxiety, and stress were associated with suicidal ideation, loss aversion was not (cross-sectional model: *P* = 0.092; Prospective model: *P* = 0.390). This suggests that the concept of loss aversion may be useful in understanding the transition from suicidal thoughts to attempts.

**Conclusion:**

This and previous studies suggest that altered decision-making is involved in suicide attempts. In our study, we show the involvement of loss aversion in particular, and propose that individuals high in loss aversion are discouraged from carrying out the suicide attempt because of a greater focus on the negative consequences of the decision.

## Introduction

Suicide and suicidal behavior is one of the leading causes of mortality and morbidity worldwide, making it a serious and significant public health problem (1). Perhaps, the most salient stressors implicated in suicidal behaviors are mental illnesses. Around 40–90% of suicide attempters suffer from depression, anxiety disorders, schizophrenia, and other mental health problems, such as alexithymia (2–5). Numerous other risk factors have been identified through association studies, such as a wide range of somatic illnesses [e.g., diabetes (6, 7), malignant neoplasms (8–10), and chronic pain (11)]; social difficulties, such as family conflicts (12, 13) and bullying (14, 15); and different socioeconomic factors, such as unemployment (16) and economic recession (17); not to mention a wide range of specific stressful life-events (18). A common denominator among these risk factors is that they often lead to suffering. However, despite their clear association to suicidality, the majority of individuals displaying these, or even a combination of these risk factors, never actually attempt suicide [see, for instance, Ref. (19–21)]. An important question is thus, what separates suffering individuals who attempt suicide from those who do not?

A number of putative mechanisms have been proposed to answer this question, from various scientific domains. A subset of behavioral studies has investigated the possible effects of decision-making on suicidal behavior. Jollant and colleagues (22), for instance, used the Iowa Gambling Task (IGT), to compare the performance of attempters, depressed non-attempters, and healthy controls. This task attempts to mimic real-life decision-making (23), and involves making a number of choices between four different decks of cards. Two of the decks are disadvantageous, as they entail large wins, but even larger losses, leading to a net loss. The losses and gains in the other two decks are smaller, but lead to a larger net profit in the long run. After sampling a few cards, healthy participants usually end up favoring the advantageous decks, while participants with for instance frontal cortex lesions underperform by selecting more cards from the high-risk decks (23–25). In the study of Jollant and colleagues (22), mood disorder patients with a history of suicide attempt performed significantly worse, both when compared to healthy controls and compared to mood disorder patients without a history of suicide attempts. In subsequent IGT studies, decreased performance was also found in bipolar patients with a history of suicidal behavior compared to other patient groups (26), or in adolescents with a previous history of suicide attempts (27). Although some studies have failed to replicate the IGT findings (28–31), a meta-analysis pooling all studies showed that suicide attempters exhibited substantially reduced performance (Hedge’s *g* = −0.47) compared with control groups of varying composition (32). Altered decision-making in suicidal individuals has also been substantiated in studies using a conceptually similar task, the Cambridge Gamble Task, among older suicide attempters (33) and young adults with a history of suicide attempts (34) and those suffering from alexithymia (35). Examination of other components of decision-making, such as sunk cost bias (i.e., the continued investment in an action with a negative outcome) and delay-discounting (i.e., the preference for smaller, but immediate rewards compared to greater, but delayed rewards) and framing has also demonstrated impaired decision-making in suicidal individuals (36, 37).

However, as much as this literature supports the association between altered decision-making in suicidal behavior, the majority of these studies are cross-sectional, which makes it difficult to draw conclusions regarding the causal mechanism of altered decision-making and suicidal behavior. It is difficult to disentangle whether the impact of a suicide attempt alters decision-making, or whether the divergent decision-making leads to suicide attempts. Another problem is that, due to the complexity of the specific IGT task-paradigm, it is difficult to identify specific cognitive components that predict performance (38), which in turn could be used to characterize suicide attempters. Studies aimed at dissecting the IGT-paradigm identified several cognitive processes that are likely to drive performance. These include higher order processes involved in understanding and learning the structure of decks, including memory functioning, but also more basic features, such as impulsivity (39–42). Last, studies have found that IGT performance is influenced by participants’ sensitivity to loss frequencies and loss magnitude (43, 44) by implicating loss aversion.

Loss aversion (45, 46) is one of the most robust and ubiquitous empirical findings in the behavioral sciences (47), which entails a strong human preference for avoiding losses, rather than making equivalent gains. In other words, the threat of a potential loss is more likely to influence human decisions compared to an opportunity for an equal gain. In tasks measuring loss aversion, participants may be offered various gambles with a 50% chance to either win or lose a certain amount of money [e.g., Ref. (48, 49)]. The magnitude of potential gains and losses are then varied for each gamble, and participants are asked to indicate whether they accept or reject them. Gambles will generally be rejected unless the potential gain is around twice the potential loss. For example, gambles offering a 50% chance to win $30, but a 50% chance to lose $20, will most often be rejected, despite the expected value being a gain of $5. From an evolutionary perspective, loss aversion can be conceptualized as an automatic protective mechanism, which through biasing the decision-maker, guides him or her away from potential danger unless reward is valuable enough to warrant it.

It could be hypothesized that an individual with greater aversion to potential losses (e.g., the physical harm if the attempt fails, the sorrow afflicted on family members, etc.) would find the option of attempting suicide less advantageous. If the potential gains (i.e., the discontinuation of suffering) have a greater influence on the decision, the option of making an attempt may be perceived as something more advantageous. In this light, aversion to potential losses can be seen as a protective factor against suicide. Subsequently, individual differences in loss aversion could distinguish between suffering attempters and non-attempters.

In this study, we aimed at investigating the association between loss aversion and suicidal behavior. We used both cross-sectional and prospective analyses, to address issues of causal directionality that previous studies have not been able to. Our study is focused on a cohort of adolescents, since the suicide attempt rate in this age group is high (50). A mixed monetary gamble task (48) was used to investigate the association between individual differences in loss aversion and suicide attempts, and whether these differences may predict future suicide attempts.

## Materials and Methods

### Study Design and Sampling Procedure

The current study utilized data from a randomized controlled trial (Current Controlled Trials ISRCTN65120704) conducted in 2012–2013 as a part of the Suicide Prevention through Internet and Media-Based Mental Health Promotion (SUPREME) project. The cluster-randomized trial used questionnaires to evaluate a mental health-promoting website among adolescents recruited from 30 randomly selected state schools in seven European countries. The intervention-effect was controlled in all prospective analyses in this study.

Adolescents were recruited from predefined catchment areas in each of the seven countries: West Viru County (Estonia; 3 schools, 416 participants), Budapest District II and District XII (Hungary; 6 schools, 413 participants), Molise (Italy; 3 schools, 311 participants), Vilnius City (Lithuania; 3 schools, 240 participants), Barcelona City (Spain; 3 schools, 182 participants), Stockholm County (Sweden; 9 schools, 337 participants), and Eastern England (United Kingdom; 3 schools, 387 participants). Eligible state schools in these areas were randomly arranged into a contact order, the order in which schools were contacted and asked to participate. If a school declined, the next school on the list was contacted. If a school accepted participation, a team of researchers went to the school and presented the background, aims, goals, and procedures of the study to the pupils verbally and through consent forms. Thus, the total baseline sample consisted of 2,286 school pupils, with 56% females and a mean age of 15.8 years (SD = 0.91 years).

Evaluation questionnaires were administered in three waves; at baseline (T1), at 2 months (T2), and at 4 months (T3). The questionnaires were administered in classrooms or computer labs during normal school hours, and after completion they also received information about the intervention. The questionnaires were administered on paper, or online if the schools could provide the pupils with laptops. The attrition rate between T1 and T2 was 20% (467 pupils), and between T2 and T3 it was 13% (244 pupils). Subjects were included in the longitudinal analyses if they had data from baseline and from either follow-up wave (T2 and/or T3). Written consent was obtained from all pupils who agreed to participate (as well as their parents’, when applicable), and the study was approved by an ethics committee in all participating countries. The procedures involved in the SUPREME trial have been described in more detail elsewhere (51).

### Measurements

Participants’ levels of depression, anxiety, and stress were measured with the 42-item version of the Depression, Anxiety, and Stress Scale [DASS-42; (52)]. Scores on each subscale range between 0 and 42. Previous studies have confirmed the validity and reliability of this scale (52–55), but suggest that adolescents might differentiate less between the three factors compared to adults (56). High internal consistency was also achieved in the current sample (depression alpha = 0.93; anxiety alpha = 0.89; stress alpha = 0.91).

Suicidal ideation and attempts were measured with Paykel’s suicide scale (57). Suicide attempts was measured with the question “Have you ever tried to take your own life?” to which participants could respond “No, never,” “Yes, during the past 2 weeks,” “Yes, between 2 weeks and 1 year ago,” or “Yes, 1 year ago or earlier.” A dichotomous “lifetime suicide attempt” variable was created for T1, T2, and T3, where all affirmative answers were coded as a “suicide attempt.” Attempts at either of the follow-ups were pooled to create a variable indicating attempt at *either* T2 *or* T3 (positives at both T2 and T3 were coded as one attempt). This was done to increase the number of suicide attempts and thus power. Suicidal ideation was measured using the mean score of the four items that regard ideation on the Paykel’s suicide scale (57). The item-response was a seven-point scale from “never” to “always” (“Have you felt, during the past two weeks, that life was not worth living?,” “Have you wished, during the past two weeks, that you were dead – for instance, that you could go to sleep and not wake up?,” “During the past two weeks, have you thought of taking your life, even if you would not really do it?,” “During the past two weeks, have you reached the point where you seriously considered taking your life or perhaps made plans how you would go about doing it?”). For the prospective analyses, participants’ pooled average at T2 and T3 was used. Individuals with missing values on either attempts or suicidal ideation at T1 or both follow-ups were excluded from the analyses. If they were missing on one follow-up only, data from the other follow-up was used.

**Figure 1:**
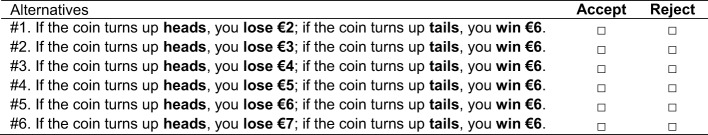
Questionnaire item used to measure individual-level loss aversion. Imagine that a person wants to make a bet with you. He flips a coin, and if it turns up heads, you lose a certain amount of money, if it turns up tails, you win a certain amount. Which of the following offers do you accept?

Loss aversion at an individual level was measured using a mixed monetary gamble task (48), shown in Figure 1 (when translating the questionnaire, the currency was adapted to each country so that the absolute amount of money would be approximately the same across settings). The potential gain always remained €6, but the potential losses were increased from €2 to €7, yielding a successively decreasing expected value for each gamble. A participant’s loss aversion score was then defined as 0 minus the highest accepted gamble, thus producing a continuous variable with a score rage of −6 to 0, where a lower score indicates lower loss aversion.

### Data Analysis

Independent samples *t*-tests and Chi-square tests were used to investigate sex differences in mental health, suicidality and loss aversion, and *t*-tests were used to examine differences in loss aversion between suicide attempters and non-attempters. A standard multiple regression was also calculated to examine how depression, anxiety, and stress (controlling for sex and intervention) was associated with loss aversion scores.

The main analyses consisted of one cross-sectional and one prospective (longitudinal) hierarchical binary logistic regression, where a life-time report of suicide attempt was used as the outcome variable (yes/no). The first model included only control variables: sex, depression, anxiety, and stress scores at T1, after which loss aversion scores from T1 was added in a second step to test if the model improved. In the prospective model, those who had reported a life-time attempt at T1 were excluded, and the outcome was thus incident suicide attempt at *either* T2 *or* T3 (possible intervention effects were controlled for in this model). All non-binary variables (depression, anxiety, stress, and loss aversion scores) were standardized (*Z*-transformed) before analyses to simplify the interpretation and comparison of odds ratios.

Two further main analyses were performed with the same control variables, but using suicidal ideation as the outcome. Because this variable is continuous, standard multiple regression was used in both the cross-sectional and prospective analysis. In the prospective analysis, suicidal ideation at baseline was included as a control variable.

All analyses were performed in SPSS version 23, with α = 0.05 (one-tailed on the main analyses). Missing data was treated using list-wise exclusion.

## Results

### Prevalence of Depression, Anxiety, and Stress in the Sample

Baseline depression scores could be computed for 2,245 participants and the mean score was 7.47 (SD = 8.38). The average baseline anxiety score was 6.75 (SD = 6.96; *N* = 2,248) and the average stress score was 10.11 (SD = 8.38; *N* = 2,246). *T*-tests showed that females had higher scores than males on all three sub-scales (Depression: M difference = 3.25; *d* = 0.40; anxiety: M difference = 2.13; *d* = 0.31; stress: M difference = 3.72; *d* = 0.46; all *P*-values <0.001).

### Prevalence of Suicidal Ideation and Behaviors in the Sample

Regarding suicide attempts, 156 (6.6%) participants reported a life-time attempt at baseline. When these subjects were excluded, there were 75 (3.4%) incident cases during the follow-up period. At baseline, females were more likely than males to have attempted suicide (females = 9.8%; males = 3.3%; $\chi_{1}^{2}=\text{35}.\text{99}$; *P* < 0.001; Phi = 0.13). There was no significant sex difference in prospective suicide attempts (females = 4.2%; males = 3.6%; *P* = 0.546). Regarding study drop-outs, participants who reported a lifetime suicide attempt at baseline were not less likely to participate at T2 (75.0 vs. 77.5%; *P* = 0.488), although they were less likely to participate at T3 (59.0 vs. 68.3%; $\chi_{1}^{2}=\text{5}.\text{8}0$; *P* = 0.021; Phi = 0.05). However, this effect size was small.

Most participants did not report suicidal ideation. The average score at baseline was 1.47 (SD = 1.05; *N* = 2,223), and the average score at follow-up was 1.37 (SD = 0.87; *N* = 2,025). *T*-tests showed that females had more suicidal ideation than males at both baseline (M difference = 0.32; *t*_2,219_ = 7.29; *P* < 0.001; *d* = 0.32) and follow-up (M difference = 0.22; *t*_1,956_ = 5.61; *P* < 0.001; *d* = 0.42). *T*-tests showed that participants with higher levels of suicidal ideation were less likely to participate at T2 (M difference = 0.16; *t*_2,221_ = 3.04; *P* = 0.006; *d* = 0.15) and T3 (M difference = 0.16; *t*_2,221_ = 3.22; *P* = 0.003; *d* = 0.14), but this effect size was small.

A cross-sectional standard multiple regression showed that higher depression and lower stress was associated with more suicidal ideation, but not anxiety or sex (Model: *F*_4, 2,187_ = 570.88; *P* < 0.001; $R_{adj}^{2}=0.\text{51}$; sex: *P* = 0.154; depression: β = 0.75; *P* < 0.001; anxiety: *P* = 0.068; stress: β = −0.09; *P* = 0.001). A cross-sectional logistic regression further showed that higher suicidal ideation and female sex was associated with a higher likelihood of suicide attempt (Model Omnibus test: $\chi_{2}^{2}=\text{31}0.\text{11}$; *P* < 0.001; Nagelkerke *R*^2^ = 0.33; suicidal ideation: Wald = 236.94; OR = 2.52; *P* < 0.001; sex: Wald = 11.70; OR = 0.456; *P* = 0.001).

### Loss Aversion

**Figure 2:**
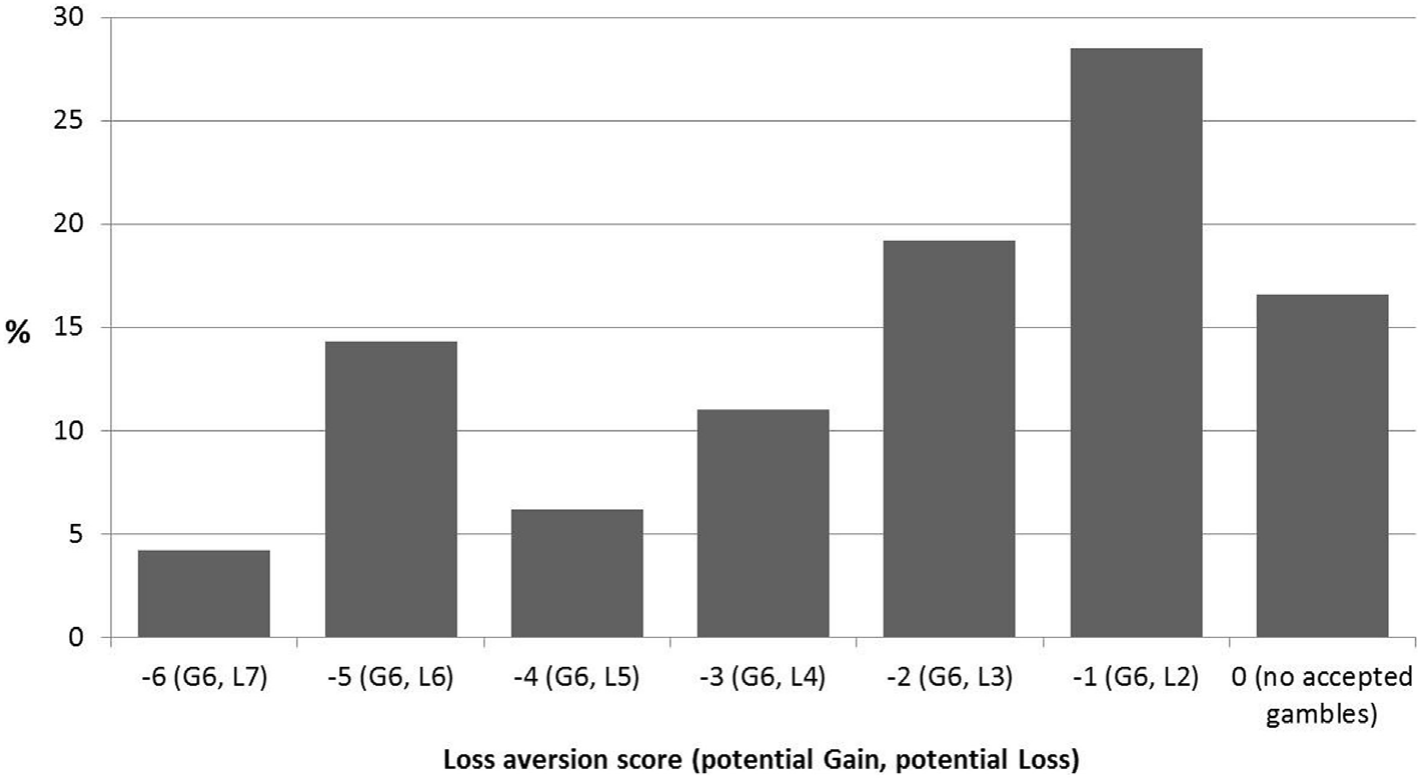
Distribution of loss aversion scores in the baseline sample (*N* = 2,203). A participant’s score is calculated as: 0 minus the highest (least profitable) accepted gamble. Thus, a score of −6 indicates very low loss aversion and a score of 0 indicates very high loss aversion. In parentheses, the potential gains (G) and losses (L) for the accepted gambles are shown. The probability of winning or losing the gamble is always 50%. Thus “G6, L7” means a 50–50% gamble with the possibility of a €6 gain and €7 loss.

Baseline loss aversion scores could be calculated for a total of 2,203 participants. The median loss aversion score was −2 (mean = −2.22; SD = 1.79), corresponding to an implied acceptable loss of €3 for a prospective gain of €6. A small minority (less than 4.2%) of the subjects accepted the gamble with the negative expected value (an implied loss of €7 for a gain of €6). Overall, the distribution of loss aversion scores in this dataset (Figure 2) compare quite well to normative data in that approximately 75% of the sample rejected gambles in which the potential gains were lower than double that of the potential loss [e.g., Ref. (48, 58)]. Compared to Gächter and colleague’s (48) sample, however, the proportion of individuals who rejected all bets was larger [16.6% in this sample compared to 1.8% in Ref. (48)]. *T*-tests showed that there was no difference in average loss aversion scores between participants who dropped out at T2 (*P* = 0.695) or T3 (*P* = 0.859), compared to participants who retained in the study. The test–retest reliability (Pearson correlation) of loss aversion was 0.54 between T1 and T2, and 0.52 between T1 and T3, and 0.63 between T2 and T3 (all *P*-values <0.001), indicating that loss aversion was relatively stable among participants over the course of 4 months.

An independent samples *t*-test showed that males had lower loss aversion scores compared to females (mean difference = 0.44; *t*_2,199_ = 5.72; *P* < 0.001; *d* = 0.24). No association was found between depression, anxiety, or stress and loss aversion scores after controlling for sex (Model: *F*_4, 2,172_ = 12.50; *P* < 0.001; $R_{adj}^{2}=0.0\text{2}$; sex: β = 0.14, *P* < 0.001; depression: *P* = 0.784; anxiety: *P* = 0.237; stress: *P* = 0.319).

Importantly, independent samples *t*-test showed that baseline suicide attempters had lower loss aversion scores compared to non-attempters (mean difference = 0.43; *t*_2,180_ = 2.59; *P* = 0.009; *d* = 0.23). Moreover, in the prospective sample with baseline suicide attempters excluded, baseline loss aversion scores were significantly lower in participants who made an attempt at follow-up, compared to those who did not (mean difference = 0.44; *t*_1,780_ = 2.02; *P* = 0.044; *d* = 0.25). There was a small cross-sectional correlation between loss aversion scores and suicidal ideation scores (*r* = −0.05; *P* = 0.016), but there was no correlation between loss aversion scores and prospective suicidal ideation (*r* = −0.03; *P* = 0.191).

### Main Analyses

The first hierarchical logistic regression was calculated to predict lifetime suicide attempts at baseline (*N* = 2,158). The first model which included sex, depression, anxiety, and stress was significant (Omnibus test: $\chi_{4}^{2}=\text{257}.\text{35}$; *P* < 0.001; Nagelkerke *R*^2^ = 0.29). Adding loss aversion scores in the next step significantly improved the model (Omnibus test: $\chi_{1}^{2}=\text{6}.\text{53}$; *P* = 0.011; Nagelkerke *R*^2^ = 0.29), with loss aversion as a significant predictor (OR = 0.79; *P* = 0.005; see Table 1). In the second hierarchical logistic regression analysis, the outcome variable was incident attempt at T2 or T3 (*N* = 1,763), and a sample where baseline suicide attempters were excluded (and intervention effects were controlled for). Similarly to the previous analysis, the first model which included only sex, baseline depression, anxiety, and stress, was significant (Omnibus test: $\chi_{5}^{2}=\text{25}.\text{32}$; *P* < 0.001; Nagelkerke *R*^2^ = 0.05). Baseline loss aversion was also a significant predictor of future suicide attempts in this model (OR = 0.81; *P* = 0.040; see Table 1), but it did not significantly improve the model as a whole (Omnibus test: $\chi_{1}^{2}=\text{3}.0\text{2}$; *P* = 0.082; Nagelkerke *R*^2^ = 0.06).

**Table 1:** Results from logistic regressions predicting suicide attempts at baseline, and prospectively during the 4-month follow-up.

Model	Predictor	Wald	OR	95% CI	*P*-value
Cross-sectional	Sex^b^	12.64	0.44	0.30–0.64	<0.001*
	Depression	42.71	2.21	1.81–2.70	<0.001*
	Anxiety	2.27	1.21	0.98–1.48	0.066
	Stress	0.51	1.11	0.87–1.43	0.238
	Loss aversion score^c^	6.63	0.79	0.67–0.92	0.005*

Prospective^a^	Sex	0.00	1.02	0.66–1.56	0.475
	Depression	8.44	1.72	1.26–2.33	0.002*
	Anxiety	1.86	1.29	0.95–1.76	0.087
	Stress	1.51	0.76	0.53–1.10	0.110
	Loss aversion score^c^	3.09	0.81	0.66–0.99	0.040*

*^a^The prospective model controlled for the effect of experimental condition*.

*^b^In sex, female sex constitutes the reference category*.

*^c^Higher loss aversion scores indicates a greater aversion to losses*.

*Sig. at P < 0.05, one-sided.

The standardized OR = 0.79 (cross-sectional) or 0.81 (prospective) for loss aversion can be interpreted as follows: an increase of one SD (i.e., 1.79 points on the six-point scale) reduces the risk of suicide attempt with approximately 20%. Expressed in absolute values (not shown in table), this corresponds to a 12% reduction in suicide risk, when loss aversion is increased with 1 point (OR = 0.87 in the cross-sectional model, and OR = 0.89 in the prospective model).

Last, standard multiple regression was carried out to test if loss aversion is associated with suicidal ideation. In the cross-sectional model, sex and the DASS-42 subscales were found to be significant predictors, but loss aversion was not (Model: *F*_5, 2,125_ = 428.75; *P* < 0.001; $R_{adj}^{2}=0.\text{5}0$; sex: β = 0.03; *P* = 0.029; depression: β = 0.74; *P* < 0.001; anxiety: β = 0.05; *P* = 0.042; stress: β = −0.09; *P* < 0.001; loss aversion: β = −0.02; *P* = 0.092). In the prospective model, only the DASS-42 subscales and baseline suicidal ideation were significant predictors, but sex and loss aversion was not (Model: *F*_7, 1,828_ = 145.14; *P* < 0.001; $R_{adj}^{2}=0.\text{36}$; sex: β = 0.01; *P* = 0.240; depression: β = 0.18; *P* < 0.001; anxiety: β = 0.07; *P* = 0.011; stress: β = −0.07; *P* = 0.029; baseline suicidal ideation score: β = 0.44; *P* < 0.001; loss aversion: β = 0.01; *P* = 0.390).

## Discussion

This study utilized a specific behavioral component of decision-making—loss aversion—to investigate its effect on suicidal behavior in a longitudinal sample of non-clinical adolescents; an age group in which the risk for attempted suicide is high. Loss aversion was hypothesized to be a protective factor against suicidal behavior. We found support for this hypothesis in that loss aversion was significantly lower among attempters compared to non-attempters, and this association remained significant even when controlling for mental health (depression anxiety and stress) and sex. Similar results were found in prospective analyses. Here, participants with no history of suicide attempts at baseline were selected, and an association was found between loss aversion scores at baseline, and attempts carried out in the subsequent 4 months (also controlling for sex and mental health). The prospective analyses strengthen the idea that altered decision-making, in particular loss aversion, is a precedent to attempt suicide, rather than being the result physical or psychological trauma followed by the attempt.

The results of our study are consistent with previous studies that implicate altered decision-making among suicide attempters [e.g., Ref. (59)]. A large body of literature reports that suicide attempters have decreased performance in IGT (32). A number of studies have argued that a key indicator in IGT performance is sensitivity to losses, that is, a preference for decks with low frequency of losses, rather than the intention of maximizing long-term gains [e.g., Ref. (60, 61)]. The lowered levels of loss aversion associated with suicide attempts are in line with these results and may thus explain this group’s poor performance on the IGT. Interestingly, however, our results are inconsistent with a recent study investigating loss aversion in a clinical population (62). Here, depressed patients with a history of suicide attempts had significantly *increased* loss aversion compared to patients with depression, but without a history of attempts, as well as compared to healthy participants. This is difficult to reconcile with our findings, and also with the previous IGT studies, considering that an increased loss aversion would predict better IGT performance. However, it must be pointed out that the complexity of the IGT gives way for a number of mechanisms that may influence performance, and it is difficult to estimate how much influence each of the components exert.

Another component of decision-making which is often considered to be closely related to loss aversion is sunk cost fallacy. This refers to an individual’s investment in a low probability pay-off because of a previously made irrecoverable investment. Szanto and colleagues (37) found that low-lethality attempters were more susceptible to sunk costs compared to non-psychiatric controls and suicidal ideators. No difference was found between high-lethality attempters and controls. The “bad investments” can be seen as the result of an aversion to the loss represented by the sunk cost. This interpretation would entail that the attempters in Szanto et al. (37) have *high* loss aversion (opposing our findings). However, the interpretation of Szanto and colleagues (37) is that low-lethality attempters are more affected by sunk costs, as a function of their proneness to emotional reactivity and impulsivity. We believe it is possible that these affect-driven components overshadow the effect of loss aversion when investigating sunk costs. For instance, older suicide attempters have shown to exhibit impaired reward/punishment-based learning compared to non-attempters and the low-lethality group with impulsive suicide attempts has been associated with enhanced discounting of delayed rewards, as well as impulsivity (63, 64). Another explanation to the discrepant findings in our study and that of Szanto and colleagues (37) may simply be that the connection between loss aversion and sunk cost bias is overestimated in general (65).

This study utilized a behavioral measure of loss aversion, but its neural underpinnings have been investigated in previous research. Loss aversion appears to be encoded in the ventromedial prefrontal cortex, orbitofrontal cortex, the ventral striatum, insula, thalamus, and the amygdala (49, 66–68), which are areas that overlap with regions found to be impaired in attempters (69–75). Neural measures of loss aversion appear to be highly consistent with behavioral measures, as shown by Tom et al. (49) (correlation of *r* = 0.85 between neural and behavioral measures). Thus, loss aversion may be an interesting endophenotype, and putative marker for future studies on suicide attempts. Also, loss aversion is often assumed to be a relatively stable and trait-like construct (76) with some empirical evidence supporting this assumption. For instance, Glöckner and Pachur (77) found a high consistency in the magnitude of loss aversion among participants’ responses in a 1-week follow-up study. Zeisberger and colleagues (78) found high stability on an aggregate level, but instability in subset of their sample, during a 1-month follow-up. Finally, in our sample the three measures, 2 months apart during the 4-month period, correlated significantly, with repeated measures explaining about 27–40% of the variance. If loss aversion is a stable trait, not only could it be directly implicated in the decision to make a suicide attempt, it could also be hypothesized that individuals with lower levels of aversion are more often exposed to losses in life in general, which in turn may entail an indirect, long-term risk for suicidal behavior.

Previous studies suggest that suicide attempters may be oversensitive to the effect of incurred losses [e.g., Ref. (79)], and also to other types of negative feedback (80). In this light, it is important to underline that loss aversion relates to the anticipation of *potential* losses, rather than a reaction to *incurred* losses. In fact, a number of studies suggest that the effects of incurred losses, on for instance cognition or arousal, are independent of the aversion to potential losses (81, 82). It is, therefore, possible for an individual to have both a low aversion to potential losses, and at the same time a high sensitivity to incurred losses. This combination could constitute an even greater increase in suicide risk in the long term, where reduced aversion to potential losses increase an individuals’ propensity for incurring losses, which is then exacerbated by an increased negative emotional reaction.

A mechanism by which loss aversion may affect the decision to make a suicide attempt is also proposed: For a suffering individual contemplating a suicide attempt, perhaps the most obvious desire, and most important “gain,” is the discontinuation of suffering. The potential “loss” may be an injury if the attempt fails, sorrow to family members and friends if it is completed, death, and so on. In this situation, individuals with higher levels of loss aversion are protected from making an attempt, because their decisions are to a greater extent influenced by the potential losses. The aversion to these make the “proposition” less attractive and it is ultimately rejected. On the other hand, individuals with lower loss aversion are more likely to focus on immediate gains, such as the discontinuation of suffering, and may consequently carry out the act.

Interestingly, we found no association between loss aversion and suicidal thoughts, despite the high association between ideation and attempts. This finding suggests that loss aversion is to a greater extent involved in the actual decision to attempt (i.e., a direct effect), rather than the events proceeding up to the genesis of the ideation. It could be interesting to further explore the relationship between loss aversion, suicidal ideation and attempts, in order to explore if loss aversion could be used to distinguish between ideators that become attempters and those that do not.

### Limitations

Given that our sample constitutes adolescents, it may be difficult to generalize the results to other age groups. Although there is some evidence to that loss aversion is similar between adolescents and adults (83), we know that suicidal behaviors are not. Attempts are usually overrepresented in the younger populations, while completed suicide is more common in older populations (1). Finally, although a prospective design was employed in this study, the validity of causal inferences may nevertheless be threatened by unmeasured confounders.

### Conclusion

The results of this study support the involvement of loss aversion in attempted suicide, through both cross-sectional and longitudinal associations. We propose that individuals high in loss aversion are discouraged from carrying out the suicide attempt because of a greater focus on the negative consequences of the decision. There is some empirical evidence, including data presented here, for the over-time stability of loss aversion. If our findings are successfully replicated, loss aversion may be considered as a candidate for a highly measurable and specific endophenotype related to suicidal behavior.

## Ethics Statement

The protocol was approved by the ethical committees from all involved centers. All subjects gave written informed consent in accordance with the Declaration of Helsinki.

## Author Contributions

GH conceived of the study. GH, SH, and AM carried out the analyses and wrote the manuscript, to which the other authors made important intellectual contributions. The other authors were responsible for the data collection in their respective countries. All authors approved of the submitted manuscript. We would like to thank Joakim Westerlund for his valuable consultation on the different aspects of this paper.

## Conflict of Interest Statement

Author NM was employed by company Skylark Health Research Ltd. All other authors declare no competing interests.
